# Visible Light-Curable Chitosan Ink for Extrusion-Based and Vat Polymerization-Based 3D Bioprintings

**DOI:** 10.3390/polym13091382

**Published:** 2021-04-23

**Authors:** Mitsuyuki Hidaka, Masaru Kojima, Masaki Nakahata, Shinji Sakai

**Affiliations:** Division of Chemical Engineering, Department of Materials Engineering Science, Graduate School of Engineering Science, Osaka University, 1-3 Machikaneyama-cho, Toyonaka, Osaka 560-8531, Japan; hidakauo@cheng.es.osaka-u.ac.jp (M.H.); nakahata@cheng.es.osaka-u.ac.jp (M.N.)

**Keywords:** bioprinting, extrusion-based printing, vat polymerization-based printing, chitosan, photocurable material

## Abstract

Three-dimensional bioprinting has attracted much attention for biomedical applications, including wound dressing and tissue regeneration. The development of functional and easy-to-handle inks is expected to expand the applications of this technology. In this study, aqueous solutions of chitosan derivatives containing sodium persulfate (SPS) and Tris(2,2′-bipyridyl) ruthenium(II) chloride (Ru(bpy)_3_) were applied as inks for both extrusion-based and vat polymerization-based bioprinting. In both the printing systems, the curation of ink was caused by visible light irradiation. The gelation time of the solution and the mechanical properties of the resultant hydrogels could be altered by changing the concentrations of SPS and Ru(bpy)_3_. The 3D hydrogel constructs with a good shape fidelity were obtained from the chitosan inks with a composition that formed gel within 10 s. In addition, we confirmed that the chitosan hydrogels have biodegradability and antimicrobial activity. These results demonstrate the significant potential of using the visible light-curable inks containing a chitosan derivative for extrusion and vat polymerization-based bioprinting toward biomedical applications.

## 1. Introduction

Three-dimensional (3D) bioprinting is an emerging technology to fabricate hydrogel constructs containing cells and/or functional materials for biomedical applications, including wound dressings and scaffolds for cell culture [1]. Material jetting-based bioprinting, material extrusion-based bioprinting, and vat polymerization-based bioprinting are the main 3D bioprinting modalities [2,3,4,5,6,7]. Primarily, extrusion-based and vat polymerization-based bioprinting have been widely studied. To widen the application of 3D bioprinting, it is important to develop inks suitable for the fabrication processes and intended applications [8,9]. To date, varieties of polymers such as gelatin, alginate, hyaluronic acid, and chitosan, including their derivatives, have been applied as ink components for 3D bioprinting [10,11,12]. Among them, chitosan has attractive features such as antimicrobial activity, biodegradability, and biocompatibility. It is one of the most well-studied biomaterials for biomedical applications, including tissue engineering and regenerative medicine [13,14,15,16]. There are several reports about 3D bioprinting using inks containing chitosan and its derivatives [17,18,19,20]. For example, Jie et al. [18] reported 3D bioprinting using ink containing carboxymethyl chitosan, sodium alginate, and gelatin. The ink was extruded from a microneedle onto a platform and cooled down to a temperature that caused thermal gelation due to the presence of gelatin. Then, the resultant hydrogel construct was further treated with CaCl_2_ aqueous solution to induce the cross-linking of alginate. Liu et al. reported 3D bioprinting using a pre-cross-linked hydrogel ink obtained from a photo-curable chitosan derivative aqueous solution through visible light irradiation [20]. In the report, the extruded pre-cross-linked hydrogel was irradiated again with visible light to stabilize the structure by post-cross-linking. A common point of these works is the necessity of multiple steps of cross-linking to obtain the final stabilized products. The inks which enable fabrication of the final products with fewer cross-linking steps and good shape fidelity are believed to be more useful for practical application. Moreover, the pre-cross-linked hydrogel ink is not suitable for stereolithographic 3D bioprinting, which requires solution inks [21].

In this study, we aimed to demonstrate the feasibility of an aqueous solution of a chitosan derivative curable through visible light-irradiation as an ink for both extrusion-based bioprinting and vat polymerization-based bioprinting. As far as we know, there are no previous reports on chitosan-based inks suitable for both the bioprinting modalities. In addition, we aimed to demonstrate that the resultant hydrogel constructs have attractive functions for biomedical applications attributed to chitosan.

The visible light-curable inks were prepared from a derivative of chitosan possessing phenolic hydroxy (Ph) moieties (chitosan-Ph), sodium persulfate (SPS), and Tris(2,2′-bipyridyl) dichlororuthenium (II) (Ru(bpy)_3_). The cross-linking process using SPS and Ru(bpy)_3_ can be progressed with exposure to visible light and has outstanding potential for biofabrication [22]. It also has been reported that aqueous solutions of derivatives of hyaluronic acid [23] and alginate [24], both possessing Ph moieties, were cured by visible light-irradiation through the formation of cross-links between Ph moieties in the presence of SPS and Ru(bpy)_3_. Photo-induced cross-linking with SPS and Ru(bpy)_3_ has the advantage of being an easy-to-control reaction by on/off switching of photo-irradiation.

In this study, we showed that our ink was rapidly curable enough for fabricating 3D constructs by both extrusion-based bioprinting and vat polymerization-based bioprinting. Additionally, we showed that the resultant hydrogel has antimicrobial activity against Gram-positive bacteria and Gram-negative bacteria and has biodegradability. These results demonstrate the great potential for the solutions of chitosan-Ph, SPS, and Ru(bpy)_3_, as inks of 3D bioprinting for biomedical applications.

## 2. Materials and Methods

### 2.1. Materials

Chitosan (Chitosan LL, deacetylation: 80%, weight average molecular weight: 50–100 kDa) was purchased from Yaizu Suisankagaku Industry (Shizuoka, Japan). Lactobionic acid, *N*,*N*,*N*′,*N*′-Tetramethylethylenediamine (TEMED), and SPS were purchased from Wako (Tokyo, Japan). Ru(bpy)_3_·Cl_2_·6H_2_O, 1-ethyl-3-(3-dimethylaminopropyl) carbodiimide hydrochloride (EDC·HCl), and 3-(4-hydroxyphenyl) propionic acid (HPP) were purchased from Sigma-Aldrich (St. Louis, MO, United States of America (USA)), Peptide Institute (Osaka, Japan), and Tokyo Chemical Industry (Tokyo, Japan), respectively. Yatalase, with complex lytic activities of fungal cell, mainly consisting of chitinase and chitobiase activities, from *Corynebacterium* sp. OZ-21, was obtained from Takara Bio (Shiga, Japan). *Escherichia coli* OP50 was cultured in LB medium containing 0.5%(*w/v*) NaCl, 1%(*w/v*) bacto tryptone (Becton Dickinson and Company, Flanklin Lakes, NJ, USA) and bacto yeast extract (Becton Dickinson and Company). For culturing *E. coli* on an agar plate, LB medium containing 1.5%(*w/v*) agar was used. *Staphylococcus aureus* was extracted from facial skin as described in the literature [25] and cultured in BHI medium containing 3.5%(*w/v*) brain heart infusion (Nissui Pharmaceutical Co., Tokyo, Japan). For culturing *S. aureus* on an agar plate, BHI medium containing 1.5%(*w/v*) agar was used.

### 2.2. Synthesis of Chitosan-Ph

Chitosan-Ph was synthesized based on information found in the literature [26,27]. Briefly, chitosan was dissolved in 20 mM HCl at 7%(*w/v*). TEMED was poured into the solution at 2%(*w/v*). Then, the pH was adjusted to 4 with NaOH. To this solution, HPP, lactobionic acid, and EDC·HCl were added at 1.5, 0.04 and 1%(*w/v*), respectively, and stirred for 20 h at room temperature. After precipitation in acetone, the resultant polymer was rinsed with 80%(*v/v*) ethanol and 20%(*v/v*) water to remove the remaining reagents, and dried in a vacuum. The content of Ph groups in chitosan-Ph was 3.0–4.8 × 10^−4^ mol-Ph/g-chitosan-Ph, which was measured by UV-vis measurement based on the literature [28].

### 2.3. Gelation Time

Gelation time was measured based on a method described in the literature [29]. Briefly, 100 μL Chitosan-Ph solution (1%(*w/v*)) containing 1–10 mM SPS and 0.5–2 mM Ru(bpy)_3_ was poured into a well of a 48-well dish and stirred using a magnetic stirrer bar at 20 rpm. Then, the solution was irradiated with visible light (3.18 W/m^2^ @ 452 nm, Figure S1 in supplementary material). The formation of a gel state was indicated by the hindrance of magnetic stirring and swelling of the solution’s surface.

### 2.4. Viscoelastic Property of Hydrogels

The viscoelastic properties of chitosan-Ph hydrogels, prepared from 1%(*w/v*) chitosan-Ph solutions containing 1–10 mM SPS and 0.5–2 mM Ru(bpy)_3_ by irradiating with visible light (3.18 W/m^2^ @ 452 nm, Figure S1) for 20 min, were measured using a rheometer (HAAKE MARS III, Thermo Fisher Scientific, Waltham, MA, USA) equipped with a parallel plate geometry at 25 °C and at a frequency of 1.6 Hz.

### 2.5. Extrusion-Based Bioprinting

A commercially available 3D printing system (FLSUN-QQ-S, Zhengzhou Chaokuo Electronic Technology Co., Henan, China) equipped with a visible-light source (LK-5BL, EK Japan, Fukuoka, Japan) was used to print 3D hydrogel structures. The light intensity was 3.18 W/m^2^ @ 452 nm (Figure S1). The inks containing 1%(*w/v*) chitosan-Ph and 1 mM Ru(bpy)_3_ were extruded from a 21-gauge stainless needle (outer diameter: 0.81 mm, inner diameter: 0.51 mm) onto the stage, moving at 6 mm/s at room temperature. The concentration of SPS and the extrusion rate of the ink were varied in the range of 1–10 mM and 3.0–13.6 mm/s, respectively.

### 2.6. Vat Polymerization-Based Bioprinting

A commercial liquid crystal display (LCD) printer (NOVA 3D, Shenzhen Nova Intelligent Technology Co., Shenzhen, China) was used to print a 3D chitosan structure. The ink containing 1%(*w/v*) chitosan-Ph, 7 mM SPS, and 2 mM Ru(bpy)_3_ was poured into a transparent plastic vat and exposed to visible light with an intensity of 0.14 W/m^2^ @ 405 nm (Figure S2 in Supplementary Material). The thickness and light irradiation time for each hydrogel layer deposition was set at 50 µm and 8 s. Rectangular structures with line and space patterns were printed to evaluate the resolution.

### 2.7. Chitosan-Ph Hydrogel Swelling

A disk of chitosan-Ph hydrogel (thickness, 1 mm; diameter, 7 mm) was printed by extrusion-based bioprinting using the ink containing 1%(*w/v*) chitosan-Ph, 4 mM SPS and 1 mM Ru(bpy)_3_. The resultant hydrogel disk was soaked in PBS and incubated in an incubator at 37 °C. The degree of swelling was evaluated from the change in diameter of the structure. Measurements were performed on five structures for 5 days.

### 2.8. Chitosan-Ph Hydrogel Biodegradability

A disk of chitosan-Ph hydrogel was printed by extrusion-based bioprinting using the ink containing 1%(*w/v*) chitosan-Ph, 4 mM SPS and 1 mM Ru(bpy)_3_. Then, the resultant hydrogel disk was soaked in a solution containing 1.9 × 10^−2^ U/mL yatalase and kept at 37 °C. The change in shape of the hydrogel disk was observed for 60 min.

### 2.9. Chitosan-Ph Antimicrobial Activity

Antimicrobial activity against photo-cured chitosan-Ph hydrogel was evaluated using Gram-negative bacteria, *E. coli*, and Gram-positive bacteria, *S. aureus*. These bacteria were cultured in LB medium and HBI medium, respectively. The solution, 100 µL, containing either of the bacteria at 10^8^–10^9^ CFU/mL, was spread on an agar plate (10 cm diameter), then, 10–100 µL of solutions containing 1%(*w/v*) chitosan-Ph, 4 mM SPS and 1 mM Ru(bpy)_3_ was put onto the agar plate and exposed to visible light (3.18 W/m^2^ @ 452 nm, Figure S1) for 10 s. Subsequently, the agar plate was incubated at 37 °C overnight. As a comparison, the phenol derivative of alginate (alginate-Ph) [24] was used instead of chitosan-Ph, and its hydrogel was prepared under the same condition on the agar plate with bacteria.

## 3. Results and Discussion

### 3.1. Hydrogelation and Hydrogel Properties

**Figure 1:**
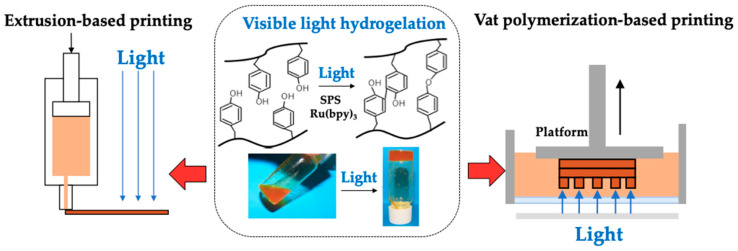
3D printing of (**center**) visible light cross-linkable chitosan-Ph solution containing SPS and Ru(bpy)_3_ through (**left**) extrusion-based printing and (**right**) vat polymerization-based printing.

**Figure 2:**
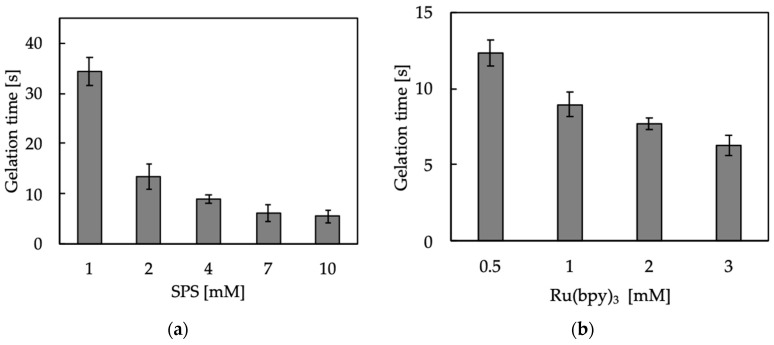
Effect of (**a**) SPS and (**b**) Ru(bpy)_3_ concentrations on gelation time of 1%(*w/v*) chitosan-Ph solutions under irradiation of visible light (3.18 W/m^2^ @ 452 nm, Figure S1). The concentrations of (**a**) Ru(bpy)_3_ and (**b**) SPS were fixed at 1 and 4 mM, respectively. Bars: mean ± SD (*n* = 10).

We first confirmed the hydrogelation of 1%(*w/v*) chitosan-Ph aqueous solution containing 2 mM of SPS and 1 mM of Ru(bpy)_3_ by visible light irradiation (3.18 W/m^2^ @ 452 nm, Figure S1) (Figure 1, center). Next, the effects of SPS and Ru(bpy)_3_ concentrations on gelation time were measured by fixing the concentrations of either SPS (4 mM) or Ru(bpy)_3_ (1 mM). The information regarding the factors affecting gelation time is important to decide the operation parameters during printing. The gelation time of chitosan-Ph decreased with an increase of SPS concentration (Figure 2a). The shortest gelation time was 5.5 ± 1.3 s. Gelation time also decreased with an increase in Ru(bpy)_3_ concentration (Figure 2b). These results corresponded with our previous report for alginate derivative possessing Ph moieties in terms of the effect on gelation time [24]. The mechanism of gelation is explained as follows [30]: (1) an electron of Ru(bpy)_3_ was excited with exposure to visible light, and the electron was donated to SPS. (2) SPS was dissociated and formed a radical. (3) The cross-linking reaction between the phenol groups on chitosan-Ph was promoted by the radical. Therefore, it was suggested that the increases in SPS and Ru(bpy)_3_ concentrations accelerated the gelation.

**Figure 3:**
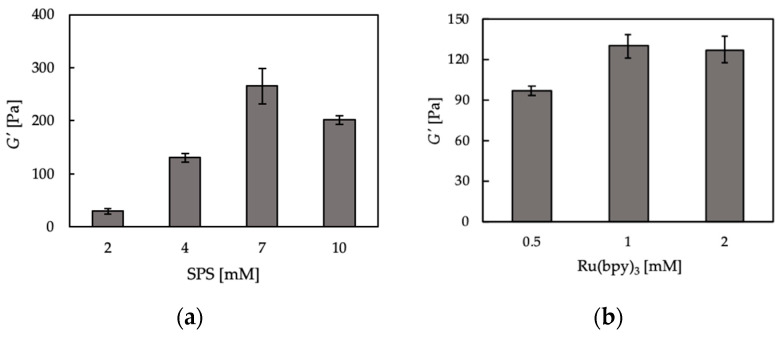
Effect of (**a**) SPS and (**b**) Ru(bpy)_3_ concentrations on storage modulus (*G’*) of 1%(*w/v*) chitosan-Ph hydrogel after irradiation with visible light (3.18 W/m^2^ @ 452 nm, Figure S1) for 1200 s. The concentrations of (**a**) Ru(bpy)_3_ and (**b**) SPS were fixed at 1 and 4 mM, respectively. The measurement was conducted a frequency of 1.6 Hz. Bars: mean ± SD (*n* = 4).

We also measured the storage modulus (*G’*) of chitosan-Ph hydrogels prepared at different SPS and Ru(bpy)_3_ concentrations. *G’* is a physical quantity expressing the stiffness of materials. As shown in Figure 3a, the storage modulus of chitosan-Ph hydrogels increased with the increase of SPS concentration (≤7 mM), but the storage modulus decreased at 10 mM compared to 7 mM. The decrease in stiffness at 10 mM SPS would be due to the degradation of the chitosan backbone through oxidization by SPS. Hong et al. [31] reported that the hyaluronic acid derivative was degraded by SPS due to the formation of persulfate free radicals. On the other hand, Ru(bpy)_3_ did not have much of an effect on the *G*’ of chitosan-Ph hydrogels (Figure 3b). As described in the cross-linking reaction mechanism above, Ru(bpy)_3_ catalyzes the cross-linking reaction, but it does not affect the storage modulus. From these results, it was suggested that SPS concentration is an important factor for 3D bioprinting of chitosan-Ph hydrogel constructs through visible light irradiation.

### 3.2. Printability of Chitosan-Ph Inks

**Figure 4:**
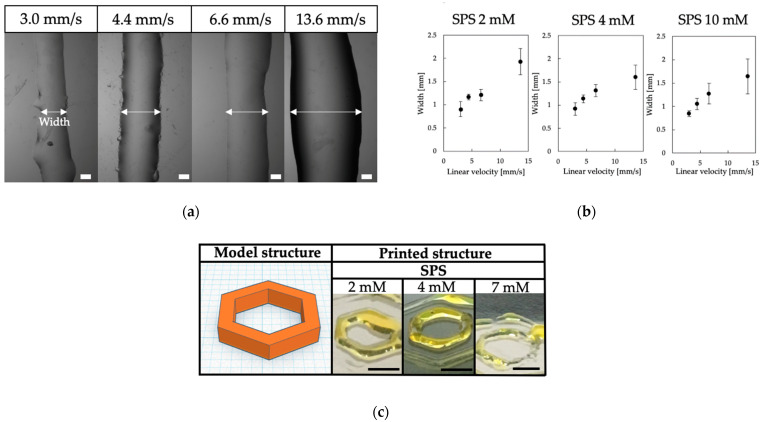
(**a**) Effect of linear velocity of ink extrusion on the width of printed line of 1%(*w/v*) chitosan-Ph ink containing 4 mM SPS and 1 mM Ru(bpy)_3_, Bars: 300 µm. (**b**) Relationship between line width and linear velocity of ink extrusion at different SPS concentration (2, 4 and 10 mM). Bars: mean ± SD (*n* = 10). (**c**) Effect of SPS concentration on the shape of printed chitosan-Ph hydrogel constructs based on a blue print (**left**) from 1%(*w/v*) chitosan-Ph ink containing 1 mM Ru(bpy)_3_ and 2–7 mM of SPS. Bars: 5 mm.

Based on the results of the effects of SPS and Ru(bpy)_3_ concentrations on the gelation of chitosan-Ph solutions and properties of resultant hydrogels, we studied the printability of chitosan-Ph inks. We first investigated the relationship between the linear velocity of ink extrusion and line width. The stable printing was achieved at 4.4 mm/s of linear velocity and 6 mm/s of stage speed with a 21-gauge nozzle (outer diameter: 0.81 mm, inner diameter: 0.51 mm) (Figure 4a). The slower linear velocity (<4.4 mm/s) caused unstable ink ejection because the flow rate of ink was not enough against the stage speed. The faster velocity (≥6.6 mm/s) caused unstable gelation due to an excess amount of ink (Figure 4b). Next, the effect of SPS concentration on printability was evaluated at a 4.4 mm/s linear velocity of ink extrusion. Higher SPS concentration (>2 mM) enabled high printability, as shown in Figure 4c. However, nozzle clogging occurred at 7 mM SPS after a while of the printing process, making printability worse. The clogging was caused by the occurrence of too rapid gelation.

**Figure 5:**
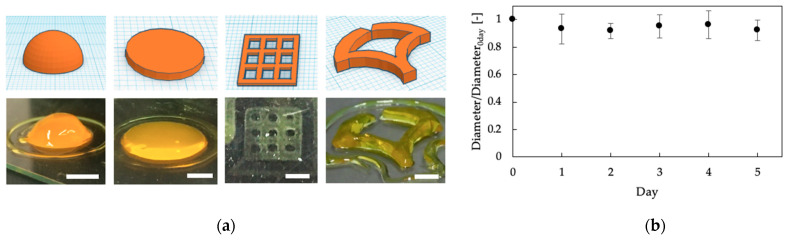
(**a**) 3D printed constructs (**bottom**) based on blueprints (**top**) by extruding 1%(*w/v*) chitosan-Ph ink containing 4 mM SPS and 1 mM Ru(bpy)_3_. Bars: 5 mm. (**b**) Change in diameter of printed disk in PBS. Disk structure was printed by extrusion-based bioprinting using 1%(*w/v*) chitosan-Ph, 4 mM SPS and 1 mM Ru(bpy)_3_. Bars: mean ± SD (*n* = 5).

Based on these results, we printed several hydrogel constructs under the following conditions: 4 mM SPS, 1 mM Ru(bpy)_3_, and 4.4 mm/s linear velocity of ink extrusion. Hemisphere, disk, and grid structures could be printed (Figure 5a). The printed hydrogel was stable in a PBS solution during the 5 days of study (Figure 5b). The stability of polymer-Ph hydrogel in the solution corresponded to the results previously reported [28,29]. For further improvement of the resolution, dye or brightener may be required [24]. There are several reports about bioprinting using inks containing chitosan [17,18,19,20]. In these previous studies, multiple cross-linking steps were required for obtaining stabilized final constructs. Our chitosan ink was ejected as a liquid from a needle and gelated rapidly by visible light. This allowed for fewer steps in building a chitosan 3D hydrogel structure without pre and post-cross-linking processes.

**Figure 6:**
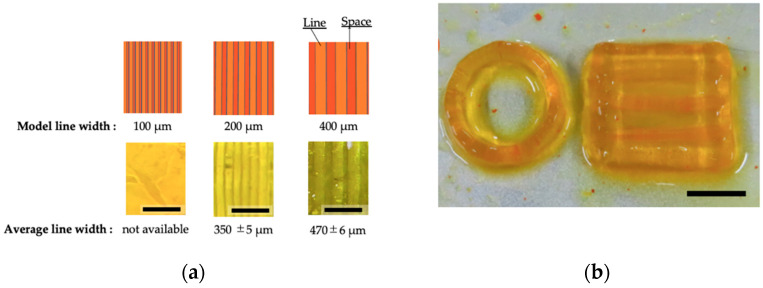
(**a**) Printed rectangular structures with line and space patterns (**bottom**) from 1%(*w/v*) chitosan-Ph ink containing 7 mM SPS and 2 mM Ru(bpy)_3_ based on blueprints (**top**). Bars: 200 µm. (**b**) Printed constructs on a platform at one time. Bar: 10 mm.

Next, we applied the chitosan-Ph solution to an LCD printer, a type of vat polymerization-based bioprinting. The resolution of the chitosan-Ph hydrogel construct was tested by building rectangular structures with different line and space patterns (Figure 6a). As shown in the figure, the patterns disappeared at 100 µm of line width. Although the size error of the printed structure was large on a submillimeter scale due to light scattering during the printing process, smaller patterns were printed with high reproducibility. Error control would be needed to achieve higher resolution printing structures during the design process or by adjusting the parameters of the printer [32]. There are several techniques of vat polymerization-based printing with light-curable material such as stereolithography appearance (SLA), digital light processing (DLP), and liquid crystal display (LCD) printing [33]. LCD printing has a high processing speed compared to extrusion-based printing because the hydrogel structure can be built layer-by-layer, while extrusion-based printing builds the structure linearly. LCD printers can also print multiple structures in parallel, unlike other methods such as material jetting-based printing and extrusion-based printing. Multiple chitosan structures were printed at one time (Figure 6b). Photo-curable chitosan-Ph solution containing SPS and Ru(bpy)_3_ enabled the effective building of 3D chitosan hydrogel constructs. This study is the first example of vat polymerization-based bioprinting using a photo-curable chitosan-Ph solution.

### 3.3. Biodegradability

**Figure 7:**
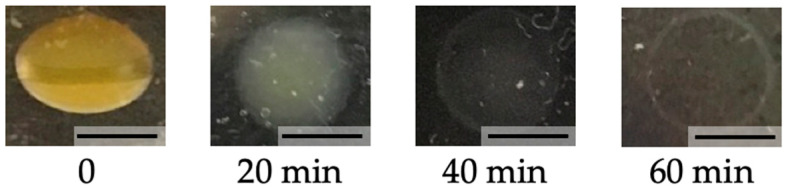
Shape change of chitosan-Ph hydrogel disk obtained from 1%(*w/v*) chitosan-Ph ink containing 4 mM SPS and 1 mM Ru(bpy)_3_ in 1.9 × 10^−2^ U/mL yatalase solution for 60 min at 37 °C. Bars: 5 mm.

We next investigated the degradability of printed chitosan-Ph hydrogel. Yatalase, as an enzyme to degrade chitosan, was used to test the biodegradability of the photo-cured chitosan-Ph hydrogel. A disk was printed at 4 mM SPS and 1 mM Ru(bpy)_3_ by extrusion-based bioprinting, and it was put in PBS containing 1.9 × 10^−2^ U/mL of yatalase. The disk was broken down almost completely in 60 min (Figure 7). We confirmed that the photo-cured chitosan-Ph hydrogel is degradable. Biodegradable material is suitable for biomedical applications because the printed material can be broken down and excreted or resorbed without removal or surgical revision [34,35].

### 3.4. Antimicrobial Activity

**Figure 8:**
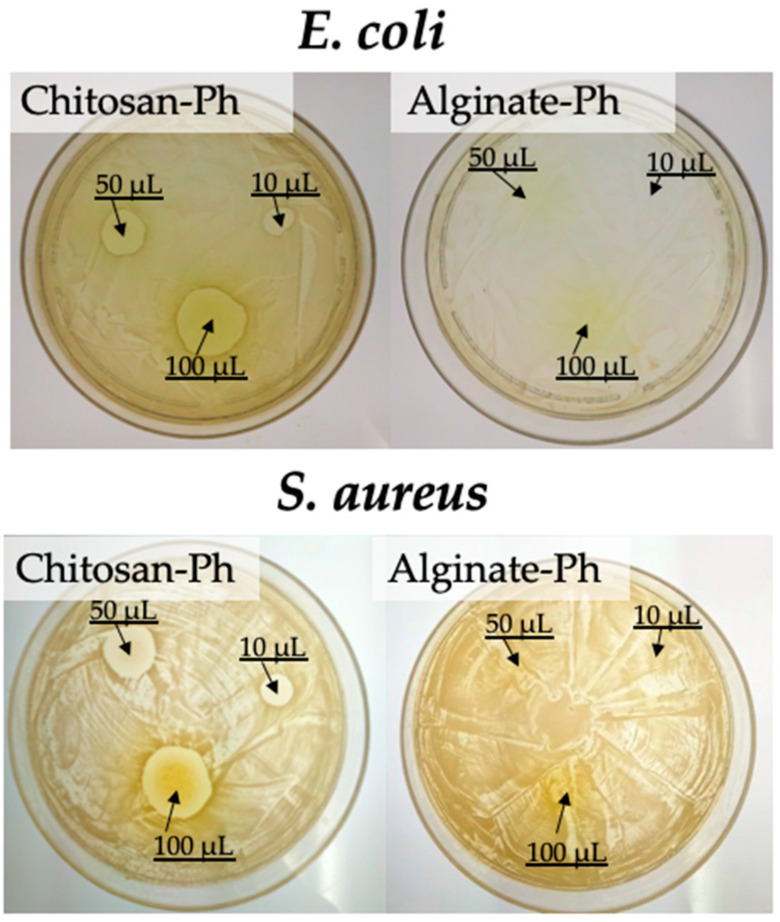
Evaluation of chitosan-Ph antimicrobial activity against (**upper**) Gram-negative bacteria, *E. coli*, on LB agar and (**bottom**) Gram-positive bacteria, *S. aureus*, on BHI agar. 10–100 µL of 1%(*w/v*) chitosan-Ph solutions containing 4 mM SPS and Ru(bpy)_3_ were spotted on agar plates with either of the bacteria and were irradiated with visible light (3.18 W/m^2^ @ 452 nm, Figure S1) for 10 s. The photographs were taken after overnight culture at 37 °C.

Finally, the antimicrobial activity of the photo-cured chitosan-Ph hydrogel was evaluated (Figure 8). Gram-negative bacteria, *E. coli*, and Gram-positive bacteria, *S. aureus*, were used for testing antimicrobial activity. The growths of both bacteria were suppressed on the area close to the chitosan-Ph hydrogel on agar. These results showed that the photo-cured chitosan-Ph hydrogel did not lose antimicrobial activity after gelation. The antimicrobial activity of chitosan has been reported in several papers [36,37,38]. The mechanism of antimicrobial activity was considered as follows [39]: Chitosan is a cationic polymer due to the protonated amino group. The bacteria cell membrane has anionic properties, and chitosan is absorbed on the surface and disrupts the activity of the bacteria. Antimicrobial activity is important, as is biodegradability, for the biomedical application of 3D bioprinting in terms of preventing bacterial infections [40,41].

## 4. Conclusions

In this study, a visible light-curable chitosan solution was applied to extrusion-based and vat polymerization-based bioprintings. The gelation time and mechanical properties of the chitosan hydrogel were controlled by altering SPS and Ru(bpy)_3_ concentrations. We confirmed that chitosan 3D structures were printed in fewer steps without pre and post- cross-linking processes by using extrusion-based bioprinting. Further, we confirmed that our chitosan hydrogel could be applied to vat polymerization-based bioprinting. Moreover, we showed that the resultant chitosan-Ph hydrogels had biodegradability and antimicrobial activity. We believe that the visible light-curable inks containing chitosan-Ph have a great potential for biomedical applications.

## Data Availability

The data that support the findings of this study are available from the corresponding author, upon reasonable request.

## Supplementary Materials

The following are available online at https://www.mdpi.com/article/10.3390/polym13091382/s1, Figure S1: Spectrum of visible light used in extrusion-based 3D printing. The spectrum was measured by an illuminance meter (CL-70F, Konica Minolta, Tokyo, Japan), Figure S2: Spectrum of visible light used in vat polymerization-based 3D printing. The spectrum was measured by an illuminance meter (CL-70F, Konica Minolta, Tokyo, Japan).
