# Supplementary material for: Visible Light-Curable Chitosan Ink for Extrusion-Based and Vat Polymerization-Based 3D Bioprintings
Source: Polymers (Basel). 2021 Apr 23;13(9):1382. doi: 10.3390/polym13091382 (PMC8122994; doi:10.3390/polym13091382)
Supplement: Supplementary file 1 [file polymers-13-01382-s001.zip › polymers-1186800-supplementary.pdf]

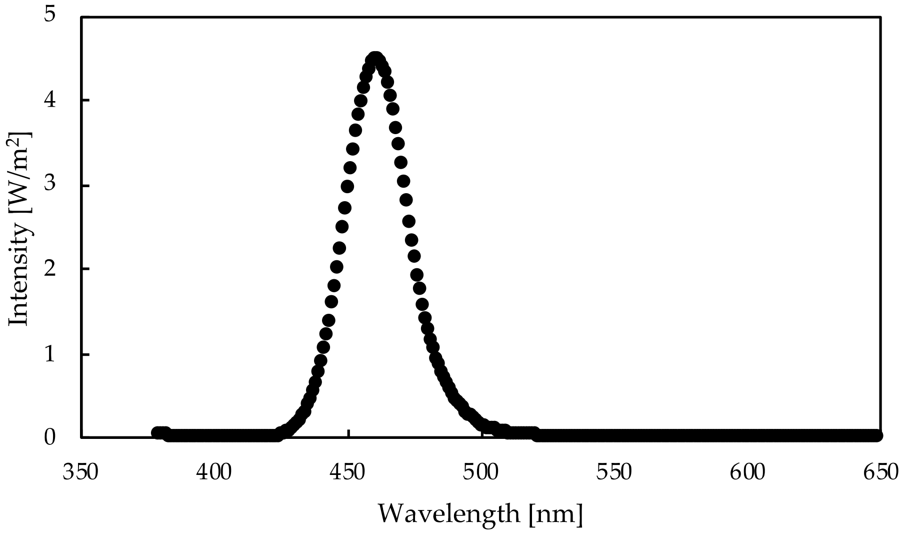

Figure S1. Spectrum of visible light used in extrusion-based 3D printing. The spectrum was measured by an illuminance meter (CL-70F, Konica Minolta, Tokyo, Japan)

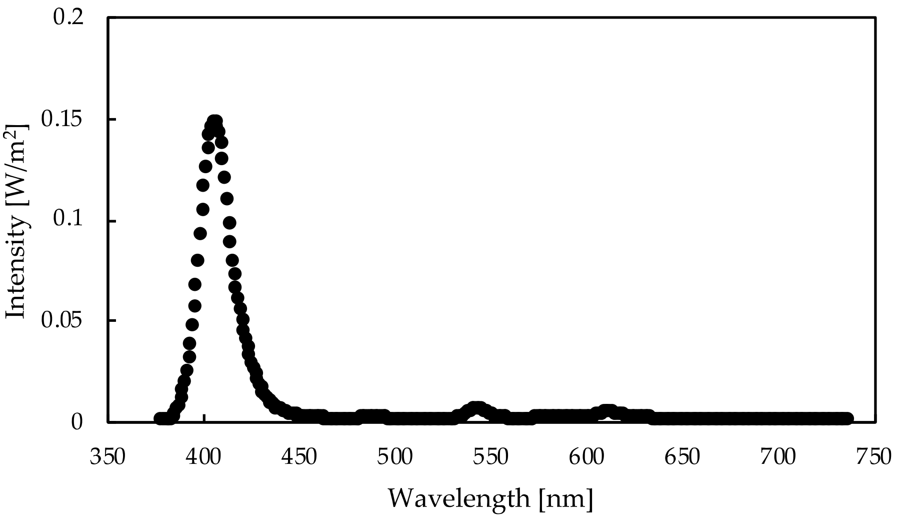

Figure S2. Spectrum of visible light used in vat polymerization-based 3D printing.The spectrum was measured by an illuminance meter (CL-70F, Konica Minolta, Tokyo, Japan).
